# Graph neural network–driven text classification for fire-door defect inspection in pre-completion construction

**DOI:** 10.1038/s41598-025-28120-4

**Published:** 2025-12-23

**Authors:** Seunghyeon Wang

**Affiliations:** Institute for Environmental Design and Engineering, University College London(UCL), London, WC1H 0NN UK

**Keywords:** Fire door defect, Text mining, Building maintenance, Deep learning, Graph neural network, Engineering, Mathematics and computing

## Abstract

Defective fire doors in apartment buildings pose significant risks by undermining fire safety measures, enabling the rapid spread of smoke and fire, and potentially endangering residents’ lives. To address this critical safety issue, this research develops and evaluates four Graph Neural Network (GNN)-based text classification models—TextGCN, TextING, TensorGCN, and BERT-GCN—for the automatic identification of fire-door defects. By systematically optimizing both general and model-specific hyperparameters, a comprehensive evaluation involving 1008 model variants was conducted using multiple performance metrics. Among these, the optimized BERT-GCN model demonstrated superior performance, achieving notable F1 scores on the test dataset across various defect categories: frame gap (91.28%), door closer adjustment (90.52%), contamination (70.75%), dent (90.21%), scratch (90.34%), sealing components (90.29%), mechanical operation components (90.29%), and others (69.99%). Overall, BERT-GCN achieved an average F1 score of 85.46%, surpassing the performance of 2,430 other evaluated text classification models. These results highlight the strong potential and effectiveness of GNN-based approaches for enhancing safety management practices in construction environments.

## Introduction

In apartment buildings, fire doors are typically installed at stairwell entrances and at the front doors of individual units. Their primary functions are to provide a safe egress route during a fire and to compartmentalize the building, limiting the spread of fire and smoke between zones^1,2^. However, it should not be assumed that fire doors remain defect-free during construction; multiple factors can compromise quality^3^. Doors may be damaged when used as access points for material transport or interior finishing, and cost-driven choices—such as inferior materials or insufficiently trained labor—can lead to installations that deviate from manufacturers’ guidelines.

Before handover, inspectors conduct visual checks and record defects in free-text form, including details such as date, contractor, and location. These descriptions are then categorized into defect types (e.g., failure to close) and damage types (e.g., dents, scratches). The resulting classifications support practical workflows: they help prioritize safety-critical repairs (e.g., fixing doors that do not latch) ahead of cosmetic issues, and they enable efficient scheduling by grouping similar tasks to reduce downtime and travel between units.

Manual classification of large volumes of fire-door defect text is time-consuming and error-prone. To the best of our knowledge, automated text-classification methods tailored specifically to fire-door defect descriptions have not been reported. Prior work in related construction contexts has often used traditional machine-learning approaches such as Naive Bayes (NB) and Support Vector Machines (SVM), which rely on relatively simple models with few parameters^4–6^. While effective for some tasks, these methods struggle to capture the nuanced linguistic patterns found in inspection narratives and defect logs^7,8^.

Recent advances in deep learning—particularly Graph Neural Networks (GNNs)—offer architectures that explicitly model semantic and structural relationships among words and phrases. By representing texts as graphs, GNNs can capture both global document context and local term interactions, extending beyond purely sequential or positional models^9^. Yet GNN variants differ in graph construction, learning regime, and computational cost, and their performance depends on task-specific design and tuning. This study therefore develops and rigorously evaluates several GNN-based methodologies for automated classification of fire-door defect text, with attention to both predictive accuracy and inference speed. The main contributions are as follows:

1. A new dataset of fire-door defect descriptions collected from apartment complexes comprising 8,786 household units.
2. An eight-class taxonomy covering seven common defect categories plus an “other” class.
3. A comprehensive evaluation of 1,008 GNN models across four architectures with systematic hyperparameter optimization.
4. An extensive benchmark against 2,430 models built from widely used machine-learning and deep-learning baselines, comparing against the best GNN.
5. A discussion of practical implications for construction management and safety operations based on the resulting findings.

## Literature review

### Traditional methods

In earlier research, traditional machine learning methods were commonly applied to text classification tasks within construction management. Salama and El-Gohary^10^ developed an approach to automatically extract compliance rules from contractual documents using NB, SVM, and Maximum Entropy (ME), achieving the highest accuracy of 82% with ME. Goh and Ubeynarayana^11^ evaluated machine learning algorithms—including SVM, Linear Regression (LR), Random Forest (RF), K-Nearest Neighbor (KNN), Decision Tree (DT), and NB—for classifying accident types from safety reports, with SVM performing best at 62% accuracy. Zhang et al.^12^ also explored accident classification using five machine learning techniques (SVM, LR, KNN, DT, NB) alongside an optimized ensemble model via Sequential Quadratic Programming (SQP), achieving an accuracy of 68% with the optimized ensemble. Further extending these methods, Ul Hassan et al.^13^ categorized project documentation into construction stages—design, construction, operation, and maintenance—using NB, SVM, LR, KNN, DT, and Artificial Neural Networks (ANN), with LR outperforming others at 94.12% accuracy.

The studies mentioned above indicate traditional machine learning approaches have demonstrated robust performance in tasks, such as defect categorization and regulatory compliance checks. However, their effectiveness diminishes in more complex scenarios, such as accident classification, primarily due to their limitations in capturing intricate contextual and semantic relationships in construction documentation^14^.

### Deep learning based methods

To address limitations in traditional methods, recent studies adopted deep learning techniques capable of capturing complex semantic contexts. Fang et al.^15^ classified near-miss incidents using models like FastText, Bi-directional Gated Recurrent Units (BiGRU), attention-based BiGRU, and Bidirectional Encoder Representations from Transformers (BERT), with BERT achieving the highest accuracy (86.91%). Similarly, Yang et al.^16^ compared CNN-based text modeling, BERT, ELECTRA, and GPT-2 for defect classification in residential facilities, with CNN-based modeling achieving 90.72% accuracy.

Further, Luo et al.^17^ performed comparative analyses between CNN and traditional methods (SVM, LR, NB) for accident classification, where CNN emerged superior with 76% accuracy. Zhong et al.^18^ conducted a comprehensive comparison among traditional (SVM, NB, LR, DT, KNN) and deep learning methods (TextCNN, TextCNN-LSTM, RCNN, Transformer) for classifying construction dispute documents, with TextCNN performing best at 65.09% accuracy. Contrastingly, Wang et al.^19^ compared DT, RF, NB, SVM, and CNN with Attention (CNN-AT) in categorizing construction defects from daily reports, finding NB unexpectedly superior at 98% accuracy. Finally, Jianan et al.^20^ evaluated deep learning techniques (Longformer-RoBERT, RoBERT, BERT) for classifying knowledge types within engineering consulting standards, with Longformer-RoBERT achieving top performance at 91.65%.

These studies suggest that deep learning methods typically achieve robust accuracy in text classification tasks due to their ability to model complex semantic information. However, traditional machine learning approaches occasionally demonstrate superior accuracy, especially in tasks with clearly defined features and simpler contextual structures, underscoring the importance of systematic comparative analyses tailored to the specific nature of each classification task.

### GNN

GNNs represent text as graphs, explicitly modeling document–term and term–term relations. Unlike sequential models (CNNs, and RNNs) and sequence-attention models (Transformers), which operate primarily over token order, GNNs aggregate information along relational edges—co-occurrence, dependency, or entity links—capturing both global document context and local lexical interactions^21^. By contrast, Transformers are naturally inductive, easy to apply to new documents without rebuilding graphs, and benefit from large-scale pretraining, but they do not exploit corpus-level relational structure as explicitly^22^.

Beyond accuracy, GNNs can be more robust than non-GNN approaches when relational structure is informative, terminology is technical and recurrent, or external structure can be injected into the graph; they also provide node- and edge-level interpretability aligned with domain concepts. These advantages have trade-offs: graph construction adds preprocessing time and memory overhead, and corpus-level graphs can complicate incremental updates and deployment^23^.

Prior studies in diverse domains—Chinese medical records^24^, factory defects^25^, and patents^26^—report strong results for GNN-based text classifiers. To the best of our knowledge, there is no prior application of GNNs to construction-defect text; given their demonstrated reliability elsewhere, this study evaluates four representative methods: TextGCN, TextING, TensorGCN, and BERT-GCN.

## Proposed approach

**Fig. 1:**
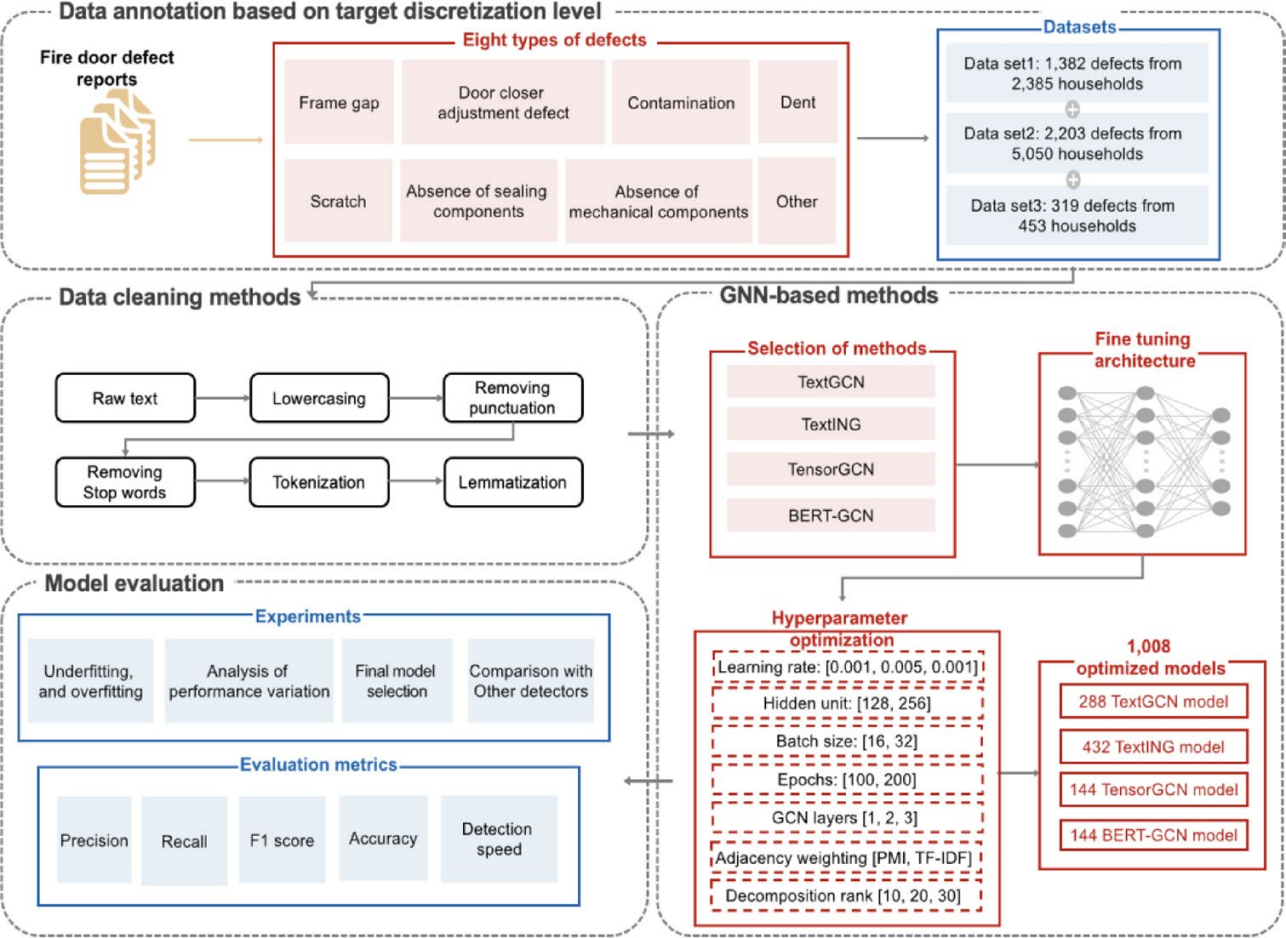
Workflow of proposed approach with demonstration.

As depicted in Fig. 1, the proposed methodology involves several key stages. Initially, real household reports documenting various fire door defects—such as frame gaps and contamination—are gathered and categorized by defect type. Following data collection, each entry within the dataset undergoes careful annotation corresponding to its specific defect category.

The dataset is then preprocessed by applying sequential text-cleaning procedures, including converting text to lowercase and conducting lemmatization. Afterwards, four distinct GNN-based models are constructed, employing fine-tuning strategies and hyperparameter optimization, yielding 1,008 different models. These developed GNN-based models are rigorously analyzed to detect potential underfitting or overfitting scenarios and subsequently evaluated alongside 2,430 additional models derived from established text classification approaches. A variety of evaluation metrics are applied to facilitate a detailed comparative analysis, with each stage elaborated in the following sections.

### Data annotation based on target discretization level

**Fig. 2:**
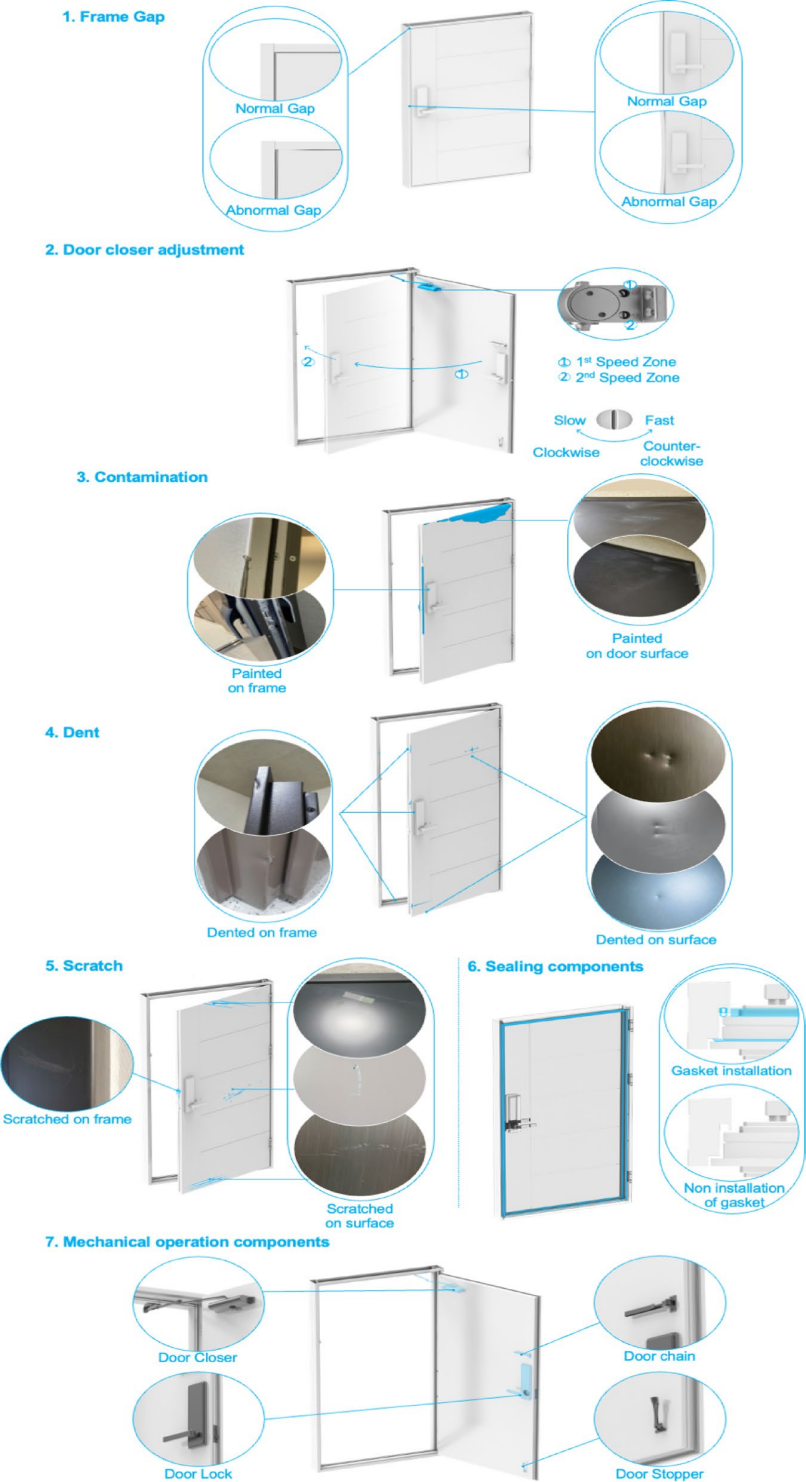
Visual examples of seven defects in fire door.

The level of annotation detail used to classify fire-door defects can vary. In this study, eight defect types are defined: (1) frame gap, (2) door-closer adjustment defect, (3) contamination, (4) dent, (5) scratch, (6) absence of sealing components, (7) absence of mechanical-operation components, and (8) others. Descriptions and example sentences for each category are provided in Table 1, and sentences are annotated accordingly. Except for “others,” each defect type is illustrated in Fig. 2.

For frame-gap defects, gaps between the door and frame commonly appear in both the horizontal and vertical directions. A door closer typically has two speed-control zones, each with an associated adjustment screw: turning the screw clockwise reduces the closing speed, while turning it counterclockwise increases it; therefore, proper closer adjustment is required. Defects such as contamination, dents, and scratches may occur on either the frame or the door surface. When sealing components are missing, this most often refers to a missing gasket. For mechanical-operation components, missing items typically include the door closer, digital door lock, hinges, or door stopper.

**Table 1:** Labeling basis on eight types of classification.

Category	Description	Example sentences
Frame gap	A condition where there is an uneven gap between the door and the door frame	Entrance 1 window (steel) Entrance gap [noise]23.12.18.Wind noise in the gap on the Entrance side. Middle door installation household
Door closer adjustment	Problems can occur due to improper adjustment of the door closer	Entrance 1 window (steel) door closure fixing defect Entrance 1 door stopper malfunction (door closes immediately)
Contamination	The presence of foreign substances like paint, oil and other contaminants on the door	Entrance 1 window (steel) lower seal damaged [Lower seal: 6/14] Entrance 1 - Entrance door sill paint stained, many scratches on stainless steel
Dent	Physical damage characterized by depressions or indentations on the door	Balcony 3 window (steel) damaged, outdoor unit room fire door damaged, Balcony 2 furniture damaged, outdoor unit room door frame dented - duplicate submission
Scratch	Surface-level damage in the form of lines or marks	Entrance 1 window (steel) door frame damaged, fire door left frame cracked in the center
Sealing components	The absence of sealing components, primarily gaskets	Balcony 3 window (steel) Fire door in outdoor unit not installed 24.01.24 Balcony 2 window not installed No rubber under protective wall
Mechanical operation components	The absence of components that directly affect door operation typically includes the door closer, digital door lock, hinges, and door stopper	Entrance 1 Window (steel) Front door not installed Front door outside stopper not installed/Photos are different
Others	Except for seven category and description, other are labelled as others	Insufficient sense of caulking, Rust on both sides of the lower part

### Data cleaning methods

**Fig. 3:**
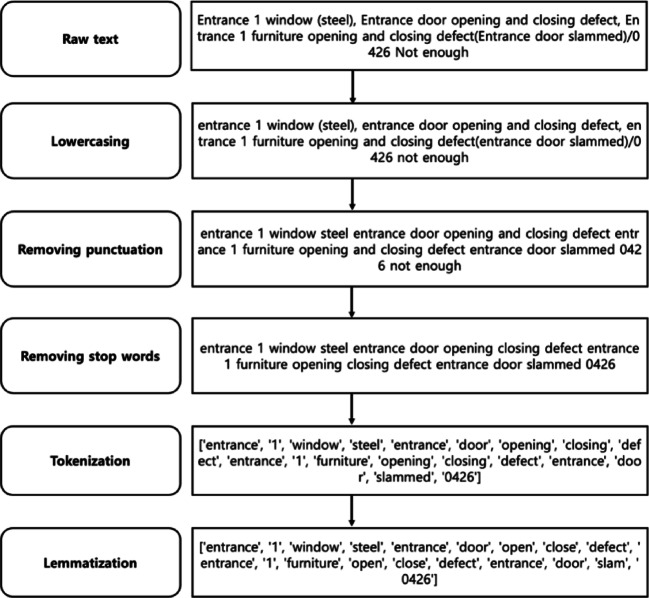
Data cleaning methods with examples.

Data cleaning is a crucial step in preparing raw text data to ensure it is consistent, relevant, and free of noise. In this research, the following data cleaning methods were applied, and how these methods transform raw text is demonstrated in the example illustrated in Fig. 3:

1. Lowercasing: This is the process of converting all characters in the text to lowercase. Lowercasing ensures that the model treats words like “Entrance” and “entrance” as the same word, avoiding redundant distinctions and simplifying the text.
2. Removing punctuation: This involves removing punctuation marks like commas, question marks, periods, etc., from the text. Punctuation often does not contribute to the meaning of the text in classification tasks, and removing it can help reduce noise, making the data cleaner and more uniform.
3. Removing stop words: Stop words are common words such as “and”, “the”, “is”, “in”, etc., that are often removed from the text because they contribute little meaning in many contexts. Removing stop words significantly reduces the size of the vocabulary and the dimensionality of the text data.
4. Tokenization: This is the process of splitting the text into individual units called tokens, which are usually words. Tokenization is essential because it transforms raw text into structured, discrete units that can be numerically represented and processed by classification algorithms.
5. Lemmatization: Lemmatization reduces words to their base or dictionary form (lemma) based on the word’s context and part of speech. For example, “opening” might be lemmatized to “open” and “slammed” to “slam” In lemmatization, words are reduced to their true root form rather than just a truncated version.

### GNN-based methods

All follow a common workflow in the selected GNN-based methods: (1) construct a graph to represent relations among textual elements; (2) learn graph embeddings; and (3) classify the embeddings with a fully connected layer^27^. In this study, TextGCN, TensorGCN, and BERT-GCN are evaluated in a transductive regime using a single corpus graph that includes train, validation, and test documents as nodes; only training labels contribute to the loss and validation/test labels are masked. TextING is evaluated inductively. Distinct characteristics are summarized in Table 2.

**Table 2:** Summary of distinct characteristics in each method.

Aspect	TextGCN	TextING	TensorGCN	BERT-GCN
Graph construction	Document-word heterogeneous graph	Word co-occurrence graph	Semantic & syntactic tensor graphs	Document-word graph with contextual embeddings
Learning type	Transductive	Inductive	Transductive	Transductive
Graph embedding approach	Graph Convolutional Networks	Gated Graph Neural Networks with GRU readout	Tensor-based Graph Convolution	Graph Convolutional Networks with transformer embeddings
Factorized Embedding	No	No	Yes	No
Computational Resources	More demanding	Moderate	More demanding	Most demanding

#### TextGCN

**Fig. 4:**
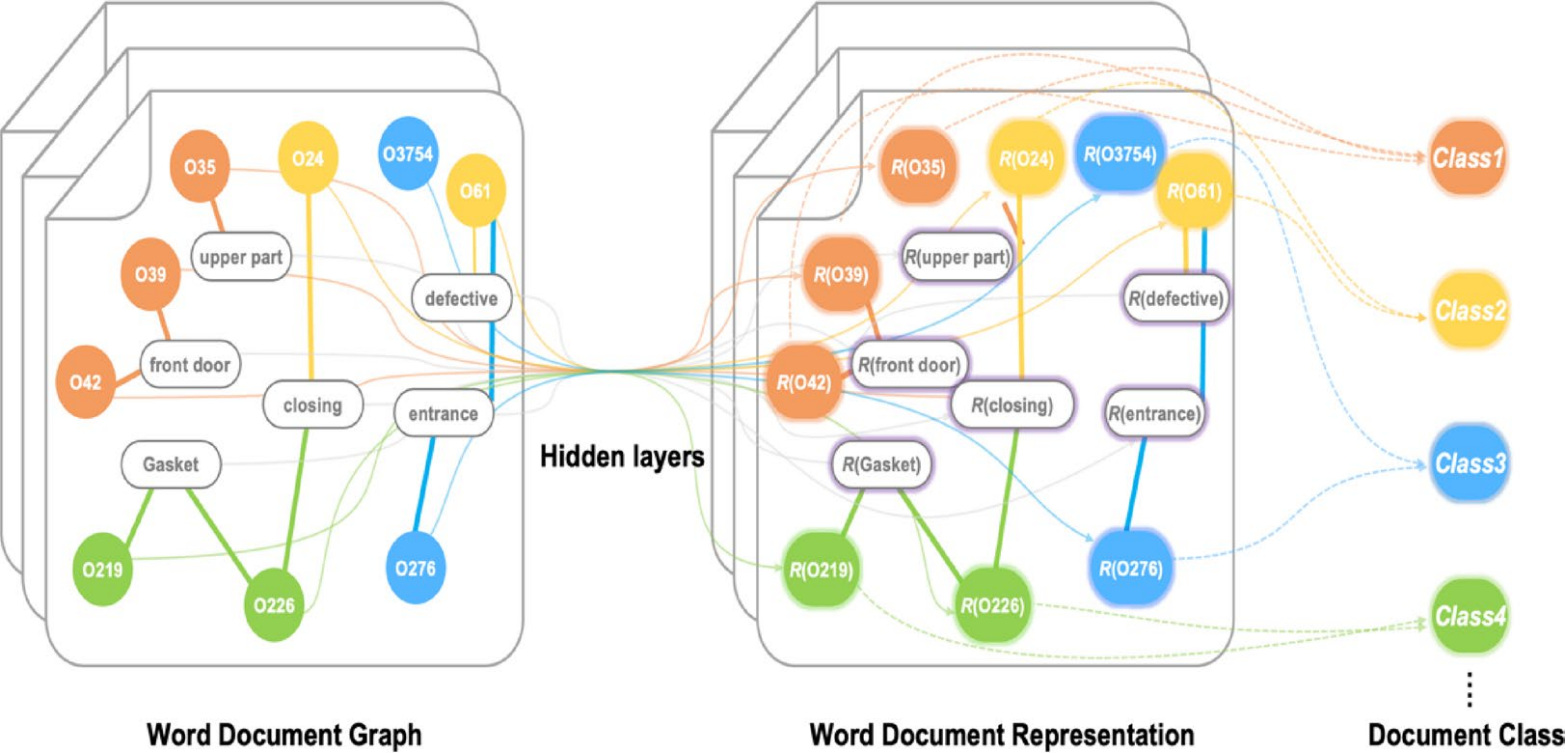
The workflow of TextGCN.

Text Graph Convolutional Network (TextGCN) (Fig. 4) constructs a heterogeneous graph that explicitly represents both documents and words as nodes, with edges capturing word-document relationships. This topology captures global document context through document–word connections and local lexical interactions through word co-occurrence. After adding self-loops and normalizing the adjacency, stacked GCN layers propagate information to produce document embeddings, which are passed to a softmax classifier^28^. The approach is attractive because it explicitly models relational structure in text, requires minimal feature engineering, and often achieves strong accuracy on corpus-level benchmarks.

#### Texting

**Fig. 5:**
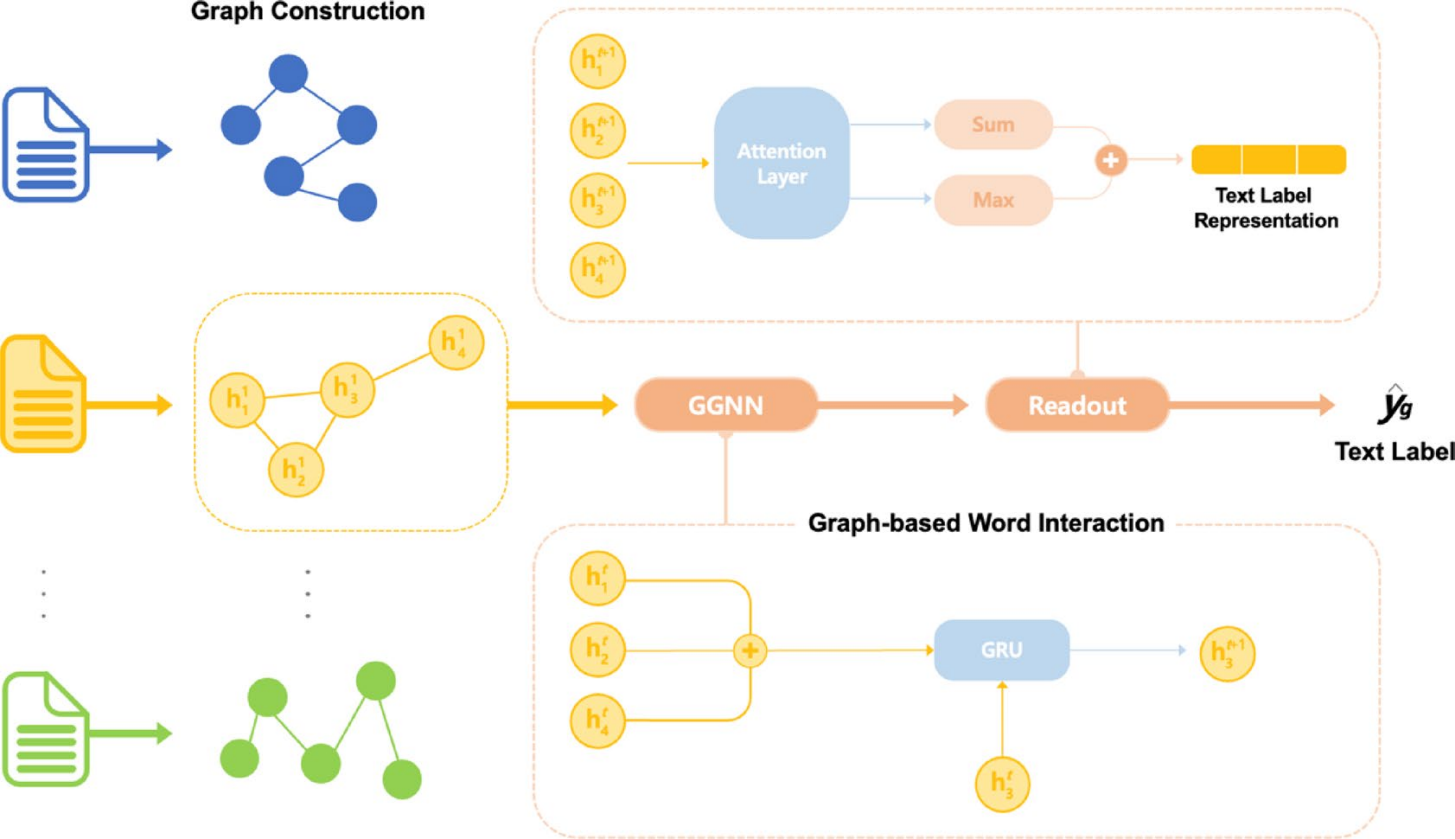
The workflow of TextING.

TextING differentiates itself by constructing a word co-occurrence graph, focusing primarily on local semantic information derived from word interactions. As shown in Fig. 5, TextING uses Gated Graph Neural Networks (GGNN) and GRU, known for their inductive learning capabilities, allowing effective generalization and application to unseen textual data^29^. This inductive learning ability provides a clear advantage for dynamic datasets or online scenarios, making TextING computationally moderate yet flexible.

#### TensorGCN

**Fig. 6:**
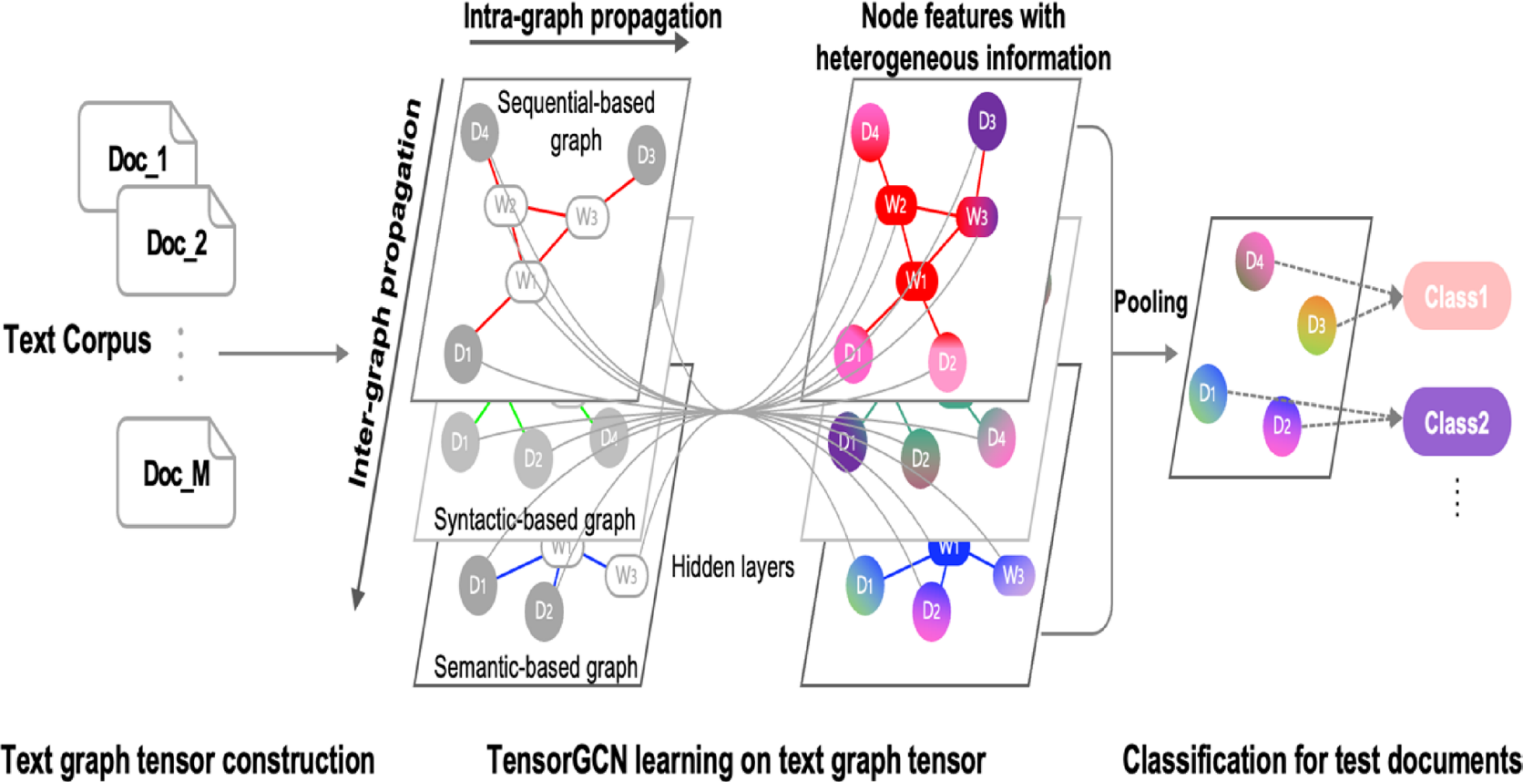
The workflow of TensorGCN.

Tensor Graph Convolutional Network (TensorGCN) distinguishes itself by constructing richer, tensor-based graphs that simultaneously capture semantic, syntactic, and sequential textual relationships, as illustrated in Fig. 6. It employs intra-graph propagation to capture detailed semantic, syntactic, and sequential interactions within each individual graph, while inter-graph propagation integrates these multiple aspects into a cohesive tensor-based representation^30^. This tensor-based graph convolution and factorized embedding strategies significantly enhance its capability to represent intricate textual relationships. However, due to the complexity introduced by handling multiple graph types and dual-propagation strategies, TensorGCN typically requires greater computational resources compared to other methods.

#### BERT-GCN

**Fig. 7:**
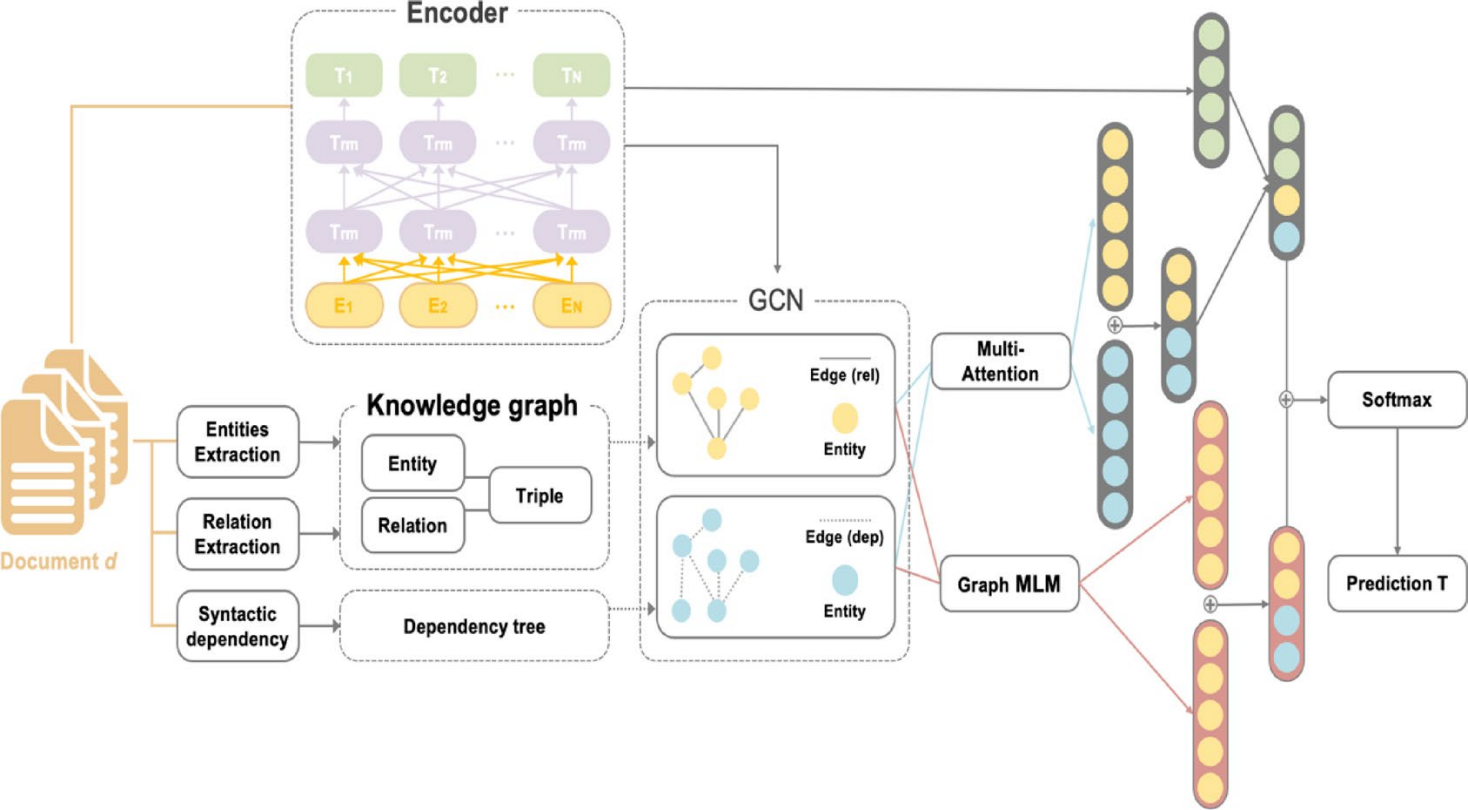
The workflow of BERT-GCN.

BERT-GCN uniquely integrates transformer-based contextual embeddings from BERT with GCN, as shown in Fig. 7. This hybrid approach first employs encoder in BERT as an encoder to obtain rich contextual embeddings from textual data, then constructs a comprehensive knowledge graph incorporating entity extraction, relation extraction, and syntactic dependencies. Subsequently, GCN leverages this graph structure to further refine node representations, supplemented by a multi-attention mechanism to capture deeper semantic interactions^31^. BERT-GCN operates transductively, processing the entire graph dataset simultaneously during embedding, which demands extensive computational resources.

### Fine tuning of architectures

After the GNN layers, the fully connected (FC) head for text classification comprises one hidden dense layer, a dropout layer for regularization, and a softmax output layer for multi-class prediction^32^. Prior studies report that using 128–256 hidden units with a dropout rate of about 0.3 yields strong accuracy and generalization^33^. In this study, the output layer contains eight neurons, matching the eight fire-door defect categories in Table 1.

### Optimization of hyperparameters

Identifying optimal hyperparameters is vital for performance, but a full sweep can be prohibitively expensive. While automated approaches—such as Bayesian optimization or random search—can reduce computation when budgets are tight or spaces are large, this work intentionally adopts grid search to provide a uniform, transparent basis for model comparison by evaluating every preset combination. Candidate ranges were drawn from prior studies, since configurations that work for related methods often transfer effectively^34^. Applying these ranges yielded 1,008 model variants. Table 3 summarizes the hyperparameters considered for each method and the resulting model counts.

**Table 3:** Detail of used hyperparameters in each method.

Algorithm	Common hyperparameters	Specific hyperparameters	Numbers of models	Best hyperparameter
TextGCN	Learning rate [0.001, 0.005, 0.01], Epochs [100, 200], Hidden units [128, 256], Batch size [16, 32]	GCN layers [1, 2, 3], Adjacency weighting [PMI, TF-IDF], Window size [5, 10]	288	Learning rate: 0.005, Epochs: 200, Hidden units: 256, Batch size: 32, GCN layers: 2, Adjacency weighting: PMI, Window size: 10
TextING		GGGN propagation layers [2, 3, 4], Aggregation [sum, mean, max], Threshold [0.1, 0.3]	432	Learning rate: 0.005, Epochs: 200, Hidden units: 256, Batch size: 32, GGGN propagation layers: 3, Aggregation: sum, Threshold: 0.3
TensorGCN		Decomposition rank [10, 20, 30], Tensor layers [1, 2]	144	Learning rate: 0.005, Epochs: 200, Hidden units: 256, Batch size: 32, Decomposition rank: 20, Tensor layers: 2
BERT-GCN		GCN layers [1, 2, 3], Adjacency weighting [PMI, TF-IDF]	144	Learning rate: 0.005, Epochs: 5, Hidden units: 256, Batch size: 32, GCN layers: 2, Adjacency weighting: PMI

### Model evaluation

#### F1 score and accuracy

The F1 score combines precision and recall into one comprehensive measure, effectively capturing the model’s ability to accurately identify relevant instances (recall) and its capacity to avoid incorrect positive classifications (precision). A high F1 score implies that the model is proficient in detecting true positives while also minimizing both false positives and false negatives, which is essential for ensuring system reliability. On the other hand, accuracy represents the ratio of correctly classified cases to the total cases evaluated, offering a direct indicator of the overall effectiveness of the model.

#### Detection speed

The detection speed refers to the computational time a model takes to process a single input instance. In this study, the detection speed is reported in terms of instances processed per second, reflecting the efficiency of each transformer-based method when analyzing textual descriptions of fire door defects.

## Experiment

### Dataset preparation

#### Raw data collection

The data used to investigate fire door defects in this study were sourced from three apartment complexes, each built by different construction companies in South Korea. Each apartment complex employed standard fire-rated steel doors. Inspectors visited a total of 8,786 households, inspecting and documenting the defects of the fire doors. As a result of the inspections, 4,212 defects were identified. The details of the households and the defects by company are outlined in Table 4. Initially, these descriptions were manually recorded in Korean by the workers and later digitized by the same workers using computers. For the purposes of this research, the Korean text was translated into English to demonstrate the context in English.

**Table 4:** Characteristics of defect datasets recorded by companies.

Construction companies	Number of households	Number of defects	Work experience of inspectors	Household Dissatisfaction (Yes/No)
J	2385	1322	More than 3 years	Yes
B	5050	2203	Less than 6 months	No
Y	453	319	Less than 1 year	No
C	925	368	Less than 6 months	No

**Fig. 8:**
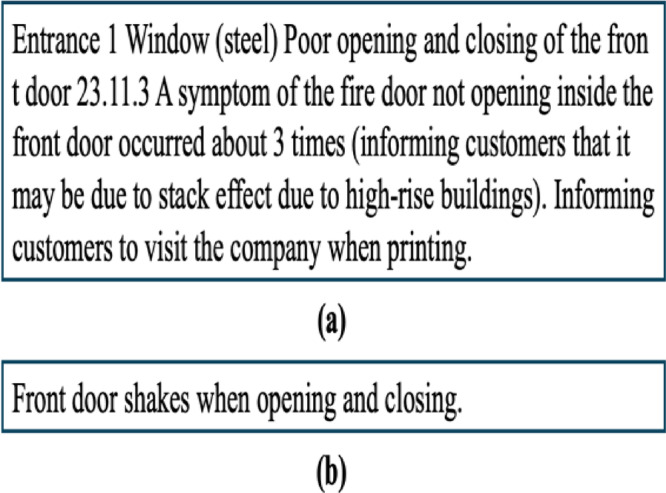
Examples of defect descriptions by work experience of inspectors.

The level of detail in the defect descriptions varied across different companies, this level of detail can be classified as different typologies in many ways. Some predominant characteristics could be easily found in the dataset, can affect the text variability by construction companies. One key factor influencing this variation was the training level of the inspectors, with some inspectors being trained to describe defects in more technical detail. Specifically, more experienced inspectors tended to use more precise technical terms. For example, Fig. 8 compares descriptions from Company J and Company B. In Fig. 8(a), the defect is described using the term “stack effect” in a detailed and technical manner. However, in Fig. 8(b), the same defect is described in a much less technical way by Company B. These inconsistencies affect token distributions and co-occurrence patterns that drive graph construction.

**Fig. 9:**
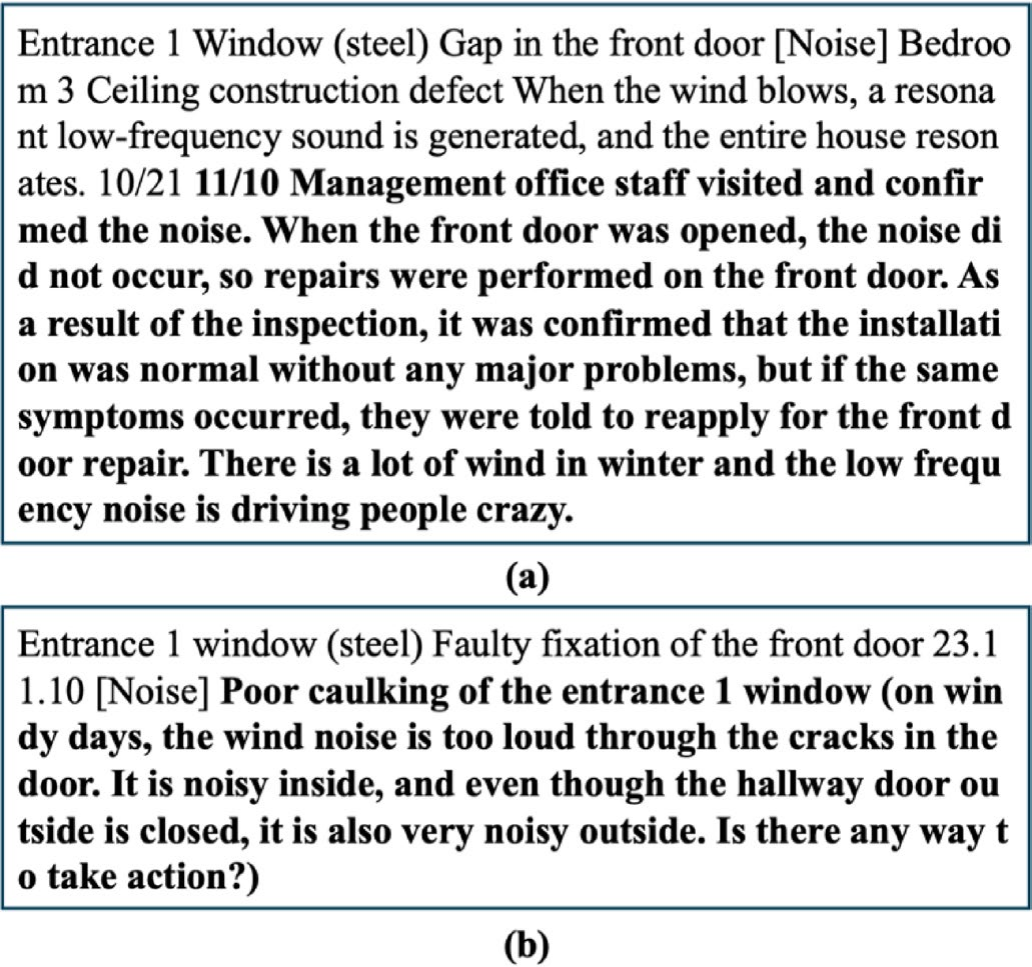
Example of variability of language.

Additionally, at Company J, residents’ dissatisfaction was logged via a mobile app. This field was retained in the text so its tokens contributed to the document–term relations used by the models, providing cues about urgency and impact without changing the labeling protocol. Figure 9 shows an example entry (dissatisfaction highlighted in bold). Variation in description detail may also reflect factors such as investigation duration, staffing levels, and inspection costs.

**Fig. 10:**
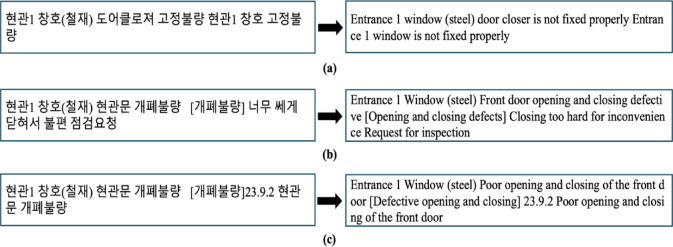
Example of description including dissatisfaction by household.

Since Korean permits diverse expressions, the defect descriptions were translated to English with attention to preserving nuance. As illustrated in Fig. 10, faults related to door opening and closing are phrased in multiple ways in Korean; this variation was maintained in the English text. The analysis informed lemmatization and synonym mapping and supported the use of graph methods that aggregate over term–term relations (co-occurrence, dependency) so semantically equivalent phrasings reinforce the same neighborhood. It also prompted clearer class definitions to reduce label drift in borderline cases.

#### Annotation

In this study, raw text records were annotated using the criteria in Table 1 to assign each entry to a fire-door defect category. To ensure annotation quality, an independent team cross-checked a sample of the texts.

#### Data split

**Fig. 11:**
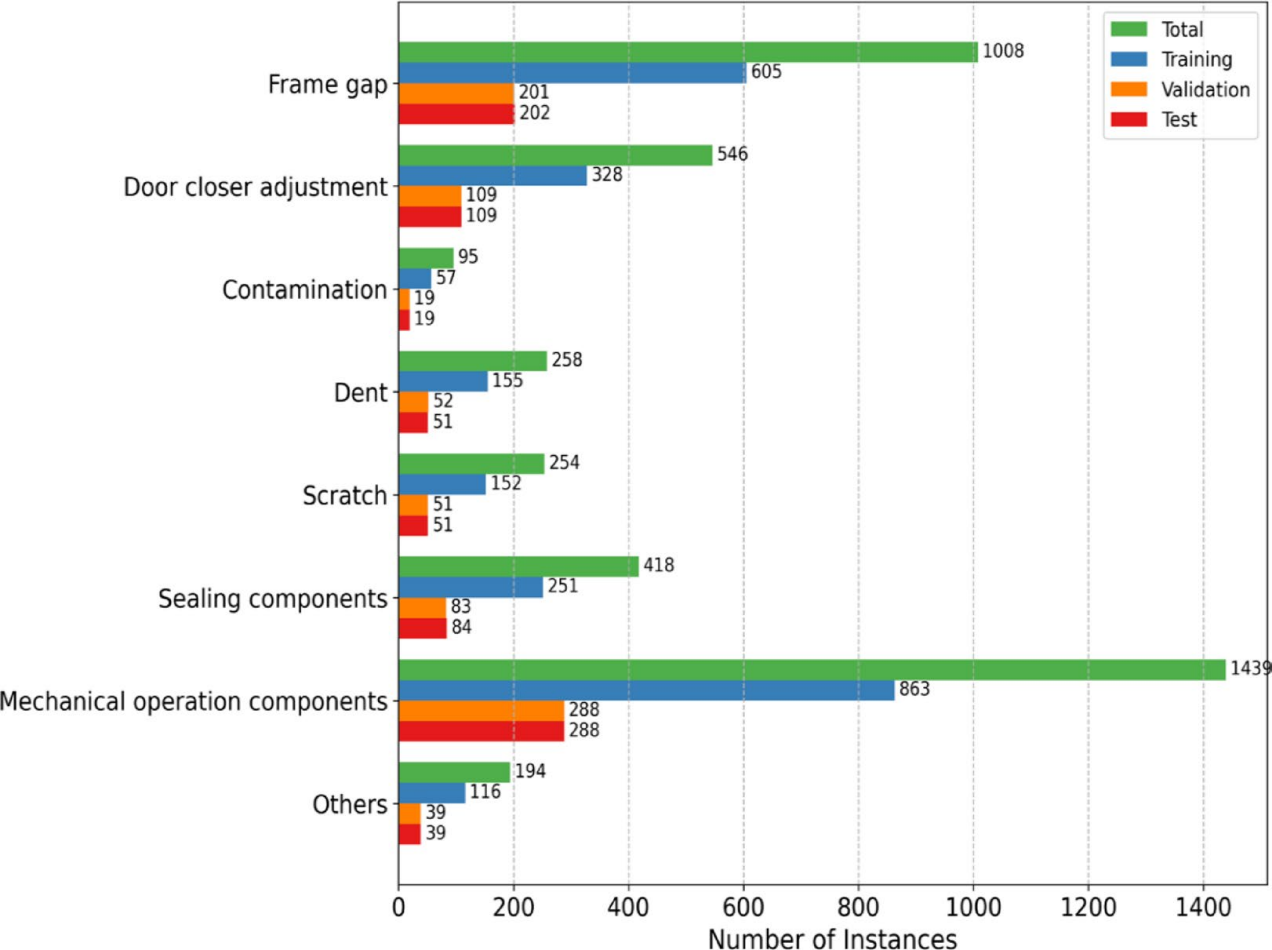
Detailed distribution of each dataset.

After annotation, the dataset containing 4,212 samples was randomly split into three subsets: a training set (60%, 2,527 samples) for developing the model, a validation set (20%, 842 samples) for evaluating and selecting the best model, and a test set (20%, 843 samples) for assessing the final model’s performance on unseen data. To address the issue of smaller classes, such as contamination and others, potentially being underrepresented or biased toward certain subsets, stratified sampling was employed. Stratified sampling ensures that the proportion of each class in the overall dataset is maintained within each subset. For example, if a particular class constitutes 5% of the total dataset, stratified sampling ensures that this class also represents approximately 5% of each subset. Figure 11 shows the detailed distribution for each dataset.

### Experimental settings

The experiments were conducted on a Windows 10 system with an Intel Core i7-7700HQ CPU (2.80 GHz, 8 threads), an NVIDIA GeForce RTX 3080 Ti GPU, and 32 GB of RAM. The GPU stack comprised NVIDIA driver 551.86, CUDA 11.8, and cuDNN 8.6. Python programming was used for implementation, leveraging Pytorch, and TensorFlow libraries to develop and execute the deep learning models. The software environment used Python 3.10.12, PyTorch 2.2.2 + cu118, and TensorFlow 2.13.1, and both frameworks were built against CUDA 11.8 and cuDNN 8.6.

## Results and discussion

In this section, a comparative analysis of the generated model results is performed. For clarity, defect types are abbreviated as follows: Frame gap as Class1, Door closer adjustment as Class2, Contamination as Class3, Dent as Class4, Scratch as Class5, Sealing components as Class6, Mechanical operation components as Class7, and Others as Class8. These abbreviations will be used throughout the section.

### Underfitting, and overfitting

**Fig. 12:**
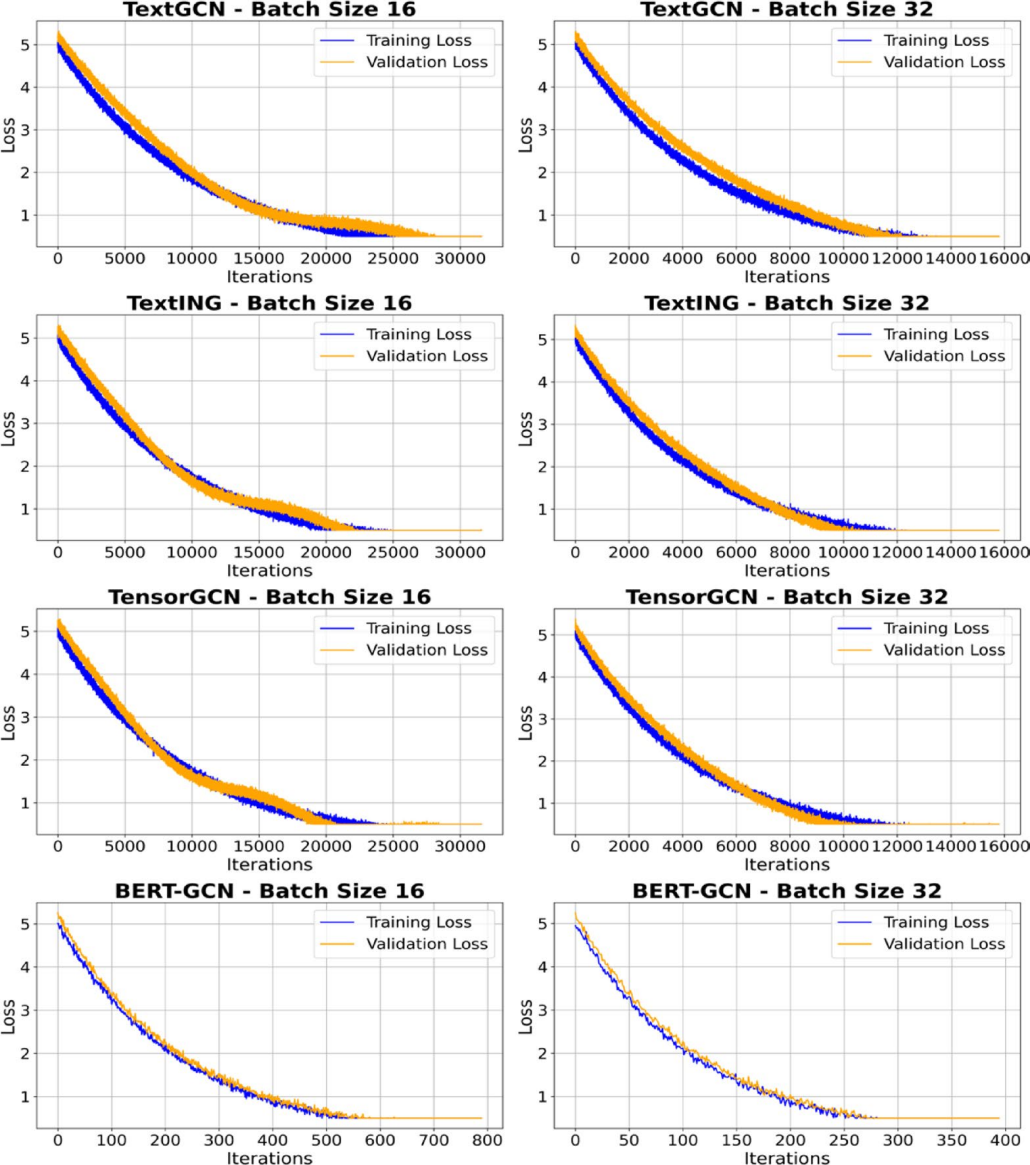
Example of training and validation loss curves by each method.

Training and validation loss curves were examined to diagnose potential underfitting or overfitting. Multiple hyperparameter settings, including batch size and number of epochs, were explored, producing 1,008 models as listed in Table 3. With 2,527 training instances, the number of batches per epoch is 158 for batch size 16 and 79 for batch size 32. Accordingly, for TextGCN, TextING, and TensorGCN trained for 200 epochs, the totals are 31,600 and 15,800 optimization steps for batch sizes 16 and 32, respectively. For BERT-GCN trained for 5 epochs, the totals are 790 steps with batch size 16 and 395 steps with batch size 32. Gradient accumulation was not used, so steps and iterations are equivalent in this setup. Example loss curves for the maximum-step setting with batch size 16 and for batch size 32 are shown in Fig. 12.

Across all models, training and validation losses decreased steadily, indicating effective learning without underfitting. The close tracking of validation loss with training loss further indicates minimal overfitting throughout training.

### Analysis of performance variation by methods

**Fig. 13:**
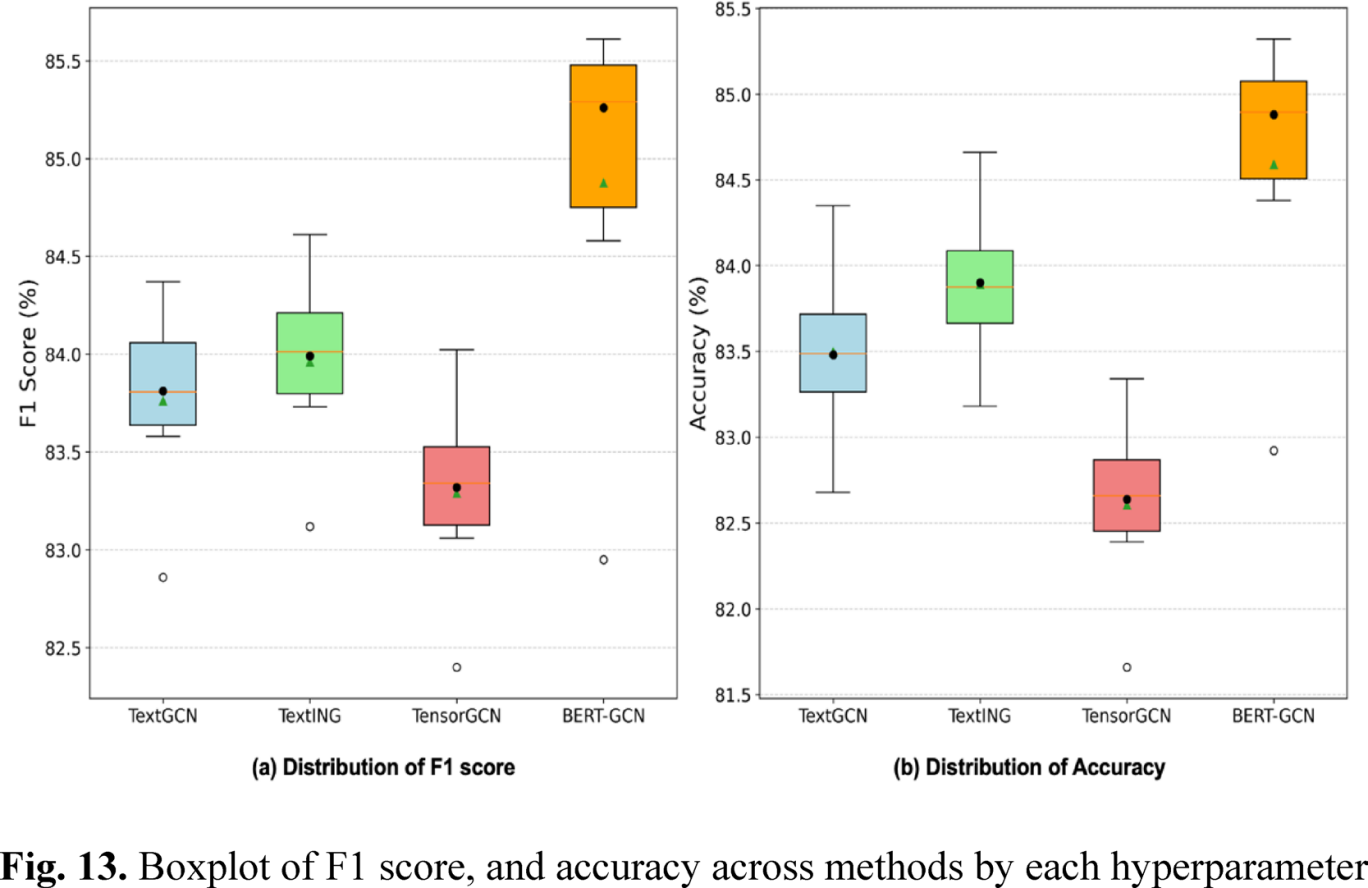
Boxplot of F1 score, and accuracy across methods by each hyperparameter.

Figure 13 presents boxplots illustrating the distribution of F1 score, and accuracy metrics for four GNN-based models by hyperparameter combinations. The boxplots illustrate the F1 score and accuracy distributions for four GNN-based models across various hyperparameter combinations. Overall, BERT-GCN achieves the highest median performance in both metrics, indicating strong predictive capability. However, its performance is not consistently superior in every hyperparameter setting, as evidenced by the overlap between its minimum values and TextING’s maximum values. TextING demonstrates good overall performance with moderate variability, closely followed by TextGCN, which shows intermediate performance with slightly higher variance. TensorGCN clearly exhibits the lowest median performance, alongside substantial variability, indicating less stability across hyperparameter configurations.

Table 5 provides detailed statistical performance of four GNN-based models. BERT-GCN demonstrates the highest mean accuracy (84.88%) and mean F1 score (85.26%), confirming superior overall performance. Although its median values are highest, performance ranges overlap slightly with TextING, indicating occasional similarity in effectiveness across hyperparameters. TextING ranks second with stable performance and moderate variability, showing consistent results across hyperparameters. TextGCN and TensorGCN exhibit lower overall performance, with TensorGCN performing the lowest in terms of both accuracy and F1 score.

**Table 5:** Statistics of F1 score and accuracy.

Model	Min	25%	Median	Mean	75%	Max	Std
F1 score							
TextGCN	82.86	83.58	83.8	83.81	84.14	84.37	0.38
TextING	83.12	83.73	84.03	83.99	84.27	84.61	0.36
TensorGCN	82.4	83.06	83.36	83.32	83.58	84.02	0.36
BERT-GCN	82.95	84.58	85.32	85.26	85.53	85.61	0.39
Accuracy							
TextGCN	82.68	83.19	83.49	83.48	83.79	84.35	0.41
TextING	83.18	83.6	83.85	83.9	84.15	84.66	0.38
TensorGCN	81.66	82.39	82.68	82.64	82.93	83.34	0.38
BERT-GCN	82.92	84.38	84.91	84.88	85.13	85.32	0.39

In addition, as shown in Table 6, BERT-GCN achieves the highest overall F1 score (85.61%), outperforming other methods across most classes (Classes 1, 2, 4, 5, 6, and 7), emphasizing its strong balance of precision and recall. However, notable weaknesses are evident in Class 3 (70.75%) and Class 8 (69.99%), suggesting challenges in capturing complex semantic or structural features. TextING ranks second (F1 score 84.61%), performing particularly better than BERT-GCN in Class 8 (72.02%), but struggling significantly in Class 3 (67.3%). TextGCN (84.37%) and TensorGCN (84.02%) perform moderately overall but similarly face challenges with Classes 3 and 8. These results highlight BERT-GCN’s superiority, yet emphasize the need to further improve models by addressing difficulties in semantically and structurally ambiguous categories.

**Table 6:** Best model in each method on validation data.

Category	Class1	Class2	Class3	Class4	Class5	Class6	Class7	Class8	Average
TextGCN									
Precision	89.94	89.04	70.9	89.15	88.96	89.20	89	69.09	84.41
Recall	89.82	89.29	70.7	89.27	88.91	88.91	88.92	68.88	84.34
F1 score	89.88	89.16	70.8	89.21	88.93	89.05	88.96	68.98	84.37
Accuracy	89.39	88.67	71.91	88.53	88.93	88.77	88.99	69.63	84.35
TextING									
Precision	90.31	89.30	67.34	89.55	89.52	89.16	89.35	71.99	84.57
Recall	90.28	89.60	67.27	89.57	89.62	89.49	89.3	72.05	84.65
F1 score	90.29	89.45	67.3	89.56	89.57	89.32	89.32	72.02	84.61
Accuracy	89.51	88.77	72.62	88.74	89.07	89.16	88.73	70.65	84.66
TensorGCN									
Precision	89.61	88.83	70.54	88.99	89.03	88.91	89.11	67.43	84.06
Recall	89.62	88.66	70.46	89.28	88.81	88.74	89.16	67.17	83.99
F1 score	89.61	88.74	70.5	89.13	88.92	88.82	89.13	67.30	84.02
Accuracy	88.73	87.97	68.28	88.36	88.03	88.51	87.98	68.90	83.34
BERT-GCN									
Precision	91.09	90.96	70.64	90.65	90.76	90.31	90.64	70.16	85.65
Recall	91.03	90.87	70.86	90.67	90.57	90.29	90.43	69.83	85.57
F1 score	91.06	90.91	70.75	90.66	90.66	90.30	90.53	69.99	85.61
Accuracy	91.02	90.44	73.07	90.45	90.33	90.11	90.21	66.96	85.32

### Final model selection

As shown in Table 6, BERT-GCN achieved the highest overall performance with the best average F1 score (85.61%) and accuracy (85.32%), clearly outperforming other evaluated methods. Conversely, TensorGCN exhibited the lowest overall performance, with an average F1 score of 84.02% and accuracy of 83.34%.

Regarding detection speed (Table 7), seconds per instance was computed as total elapsed inference time divided by the number of test samples, using batch size 32 and averaged over five runs. TensorGCN was the fastest (0.0197 s per instance), whereas BERT-GCN was the slowest (0.0267 s per instance). Despite the slower inference, the optimized BERT-GCN was selected as the final model because its higher accuracy and F1 score justified the speed–accuracy trade-off.

**Table 7:** Detection speed by GNN-based methods.

Method	Seconds per instance						
	Mean	Min	25%	50%	75%	Max	Std
TextGCN	0.0221	0.0218	0.0218	0.0223	0.0225	0.0228	0.0006
TextING	0.0229	0.0222	0.0226	0.0222	0.0233	0.0236	0.0006
TensorGCN	0.0197	0.019	0.0193	0.0199	0.0201	0.0204	0.0006
BERT-GCN	0.0267	0.0258	0.0261	0.0264	0.0272	0.0276	0.0008

### Model evaluation in test data

**Fig. 14:**
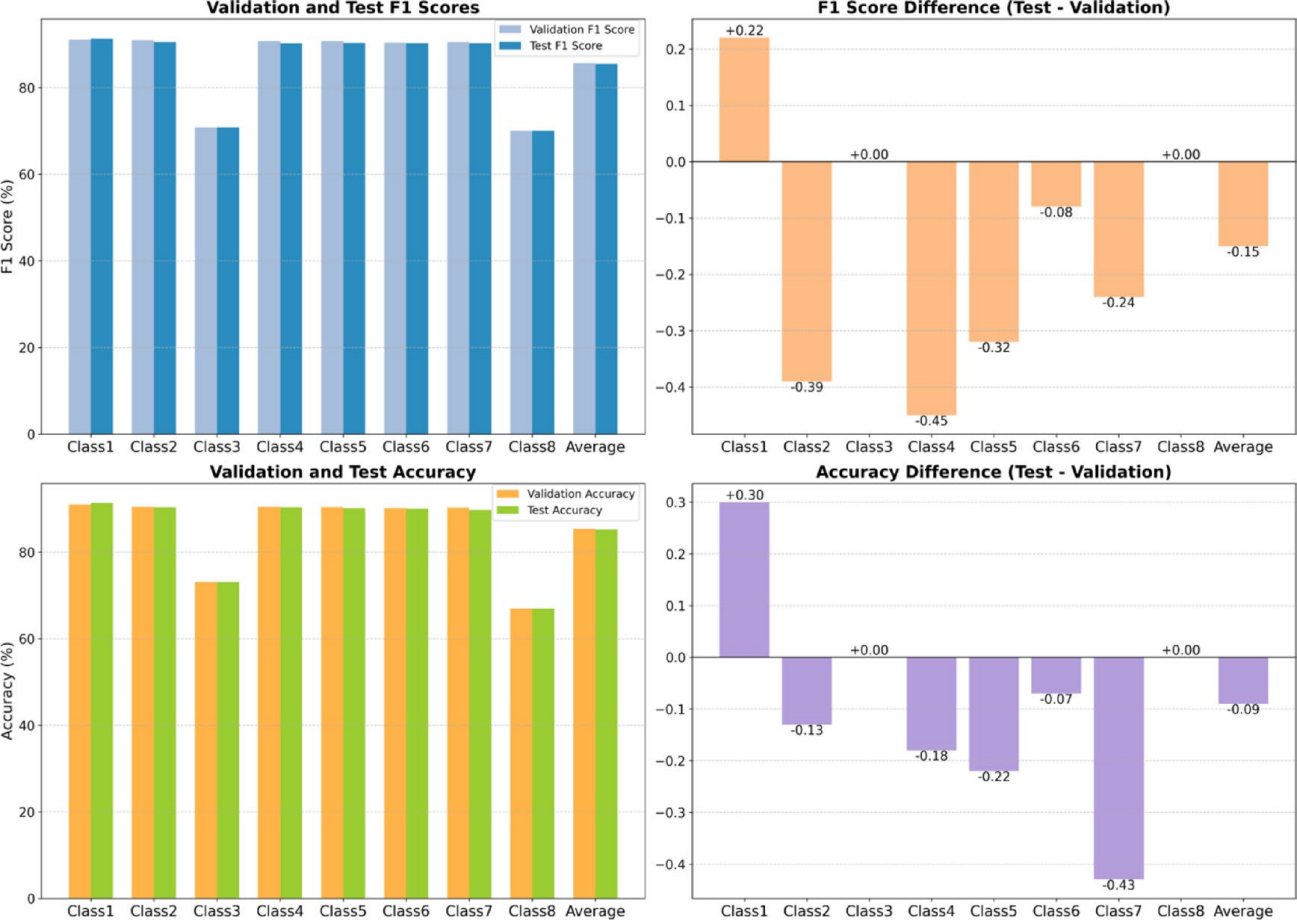
Visualization of validation and test results variation (BERT-GCN).

Table 8 compares validation and test results for the selected BERT-GCN model. Performance is stable across splits: average F1 score is 85.61% on validation and 85.46% on test, and accuracy is 85.32% on validation and 85.23% on test (Fig. 14). Class-wise results are similarly consistent for most categories (Classes 1–6). Class3 (Contamination) and Class8 (Others) remain the most challenging, exhibiting lower F1 scores than the egress-critical classes. The small validation–test differences (within ± 1%) indicate good generalization without noticeable overfitting or underfitting.

For operational protection, the stable, high performance on Class1, Class2, Class6, and Class7 supports automated triage during inspections: high-confidence predictions for these egress-critical categories can trigger same-day work orders, medium-confidence cases can be queued for rapid human review, and Class4–Class5 (cosmetic) can be grouped for planned maintenance. With the measured speed of ~ 0.026–0.027 s per instance for BERT-GCN (Table 7), near-real-time decision support on tablets is feasible.

Regarding entry-point protection oversight, class-level outputs can drive supervisory metrics and early warnings: track time-to-repair for Class1–Class2, monitor recurrence rates by contractor or floor, and generate heatmaps to reveal systematic issues (e.g., repeated closer mis-adjustment). The weaker results for Class3 and Class8 should be routed to mandatory human verification and targeted guideline refinement (e.g., tighter phrasing and synonym lists).

**Table 8:** Comparison of validation, and test results in BERT-GCN model.

Metric	Class	Validation	Test
F1 score	Class1	91.06	91.28
	Class2	90.91	90.52
	Class3	70.75	70.75
	Class4	90.66	90.21
	Class5	90.66	90.34
	Class6	90.37	90.29
	Class7	90.53	90.29
	Class8	69.99	69.99
	Average	85.61	85.46
Accuracy	Class1	91.02	91.32
	Class2	90.44	90.31
	Class3	73.07	73.07
	Class4	90.45	90.27
	Class5	90.33	90.11
	Class6	90.12	90.05
	Class7	90.21	89.78
	Class8	66.96	66.96
	Average	85.32	85.23

**Fig. 15:**
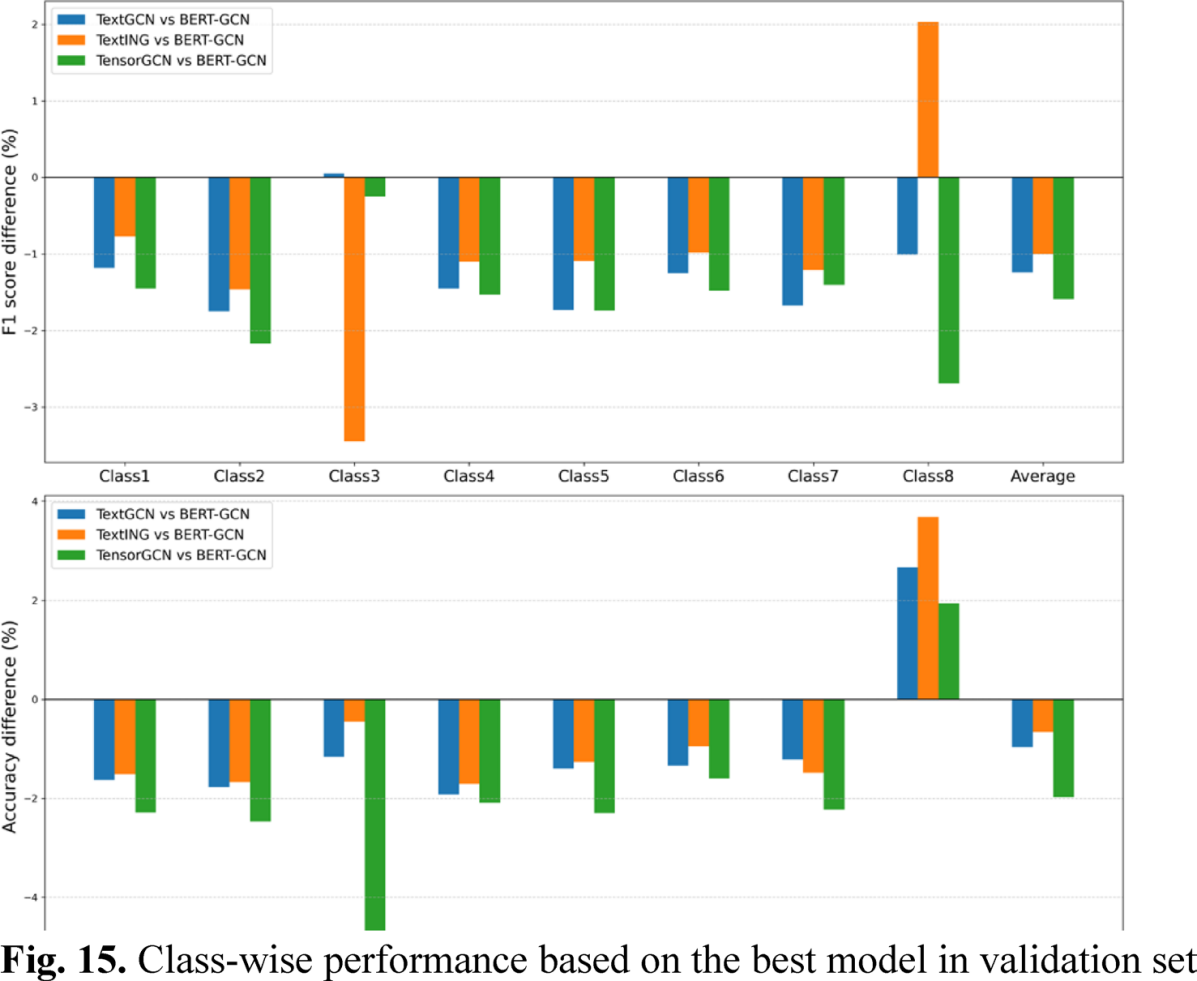
Class-wise performance based on the best model in validation set.

Figure 15 presents a comparative analysis of class-wise performance across evaluated GNN-based models (TextGCN, TextING, TensorGCN, and BERT-GCN), based on the results provided in Table 8. On average, TensorGCN shows the most substantial performance gaps relative to BERT-GCN, with negative variations reaching up to -1.98% in accuracy (85.32% vs. 83.34%) and − 1.59% in F1 score (85.61% vs. 84.02%). TextING and TextGCN display moderate performance variations, with TextING marginally outperforming TextGCN in both accuracy (+ 0.31%, 84.66% vs. 84.35%) and F1 score (+ 0.24%, 84.61% vs. 84.37%). Notably, in Class 8, the BERT-GCN model exhibits a lower F1 score (69.99%) compared to TextING (72.02%) and demonstrates the lowest accuracy (66.96%) among all models.

### Comparison with other detectors

#### Selection of other detectors

To demonstrate the superiority of the proposed BERT-GCN-based approach, comparisons were conducted with nine widely used text classification methods, including ANN, SVM, DT, RF, LR, 1D CNN, LSTM, BiLSTM, and BERT. Additionally, various vectorization techniques: Term Frequency-Inverse Document Frequency (TF-IDF), Bag-of-Words (BOW), 3-gram, Word Embedding, Word2Vec, and FastText were utilized. Hyperparameters for each method were empirically optimized through preliminary experimentation, resulting in a total of 2,430 evaluated models across all methods. Table 9 summarizes the classification methods along with their optimized hyperparameters and presents the best-performing configurations identified from the test dataset.

**Table 9:** Comparison methods with hyperparameters.

Algorithm	Vectorization method	Hyperparameter	Numbers of models	Best model
ANN	TF-IDF, BOW, 3-gram	Hidden layers: [1,2,3]; Nodes per layer: [64,128,256]; Activation: [relu, tanh]; Learning rate: [0.0001,0.001,0.01]	162	TF-IDF, Hidden layers: 2, Nodes: 128, Activation: relu, Learning rate: 0.001
SVM		C: [0.01,0.1,1,10]; Kernel: [linear, rbf]; Gamma: [scale, auto]	48	TF-IDF, C: 10, Kernel: linear, Gamma: scale
DT		Max depth: [5,10,15,None]; Criterion: [gini, entropy]; Max features: [auto, sqrt, None]	72	BOW, Max depth: None, Criterion: entropy, Max features: sqrt
RF		Number of estimators: [100,200,300]; Max depth: [5,10,15,None]; Criterion: [gini, entropy]; Max features: [auto, sqrt, None]	216	TF-IDF, Number of estimators: 300, Max depth: None, Criterion: entropy, Max features: auto
LR		C: [0.01,0.1,1,10]; Penalty: [l1,l2]; Solver: [newton-cg, lbfgs, liblinear, sag, saga]	60	TF-IDF, C: 1, Penalty: l2, Solver: saga
1D CNN	Word embeddings, Word2Vec, FastText	Conv layers: [1,2]; Filters: [64,128,256]; Kernel sizes: [3,5]; Pooling: [max, avg]; Dropout: [0.1,0.2,0.3]; Learning rate: [0.0001,0.001]; Batch size: [32,64]; Epochs: [10,20]	576	FastText, Conv layers: 2, Filters: 256, Kernel size: 3, Pooling: max, Dropout: 0.2, Learning rate: 0.0001, Batch size: 32, Epochs: 20
LSTM		LSTM layers: [1,2]; Hidden units: [64,128,256]; Embedding size: [200,300]; Dropout: [0.1,0.2,0.3]; Learning rate: [0.0001,0.001]; Batch size: [32,64]; Epochs: [10,20]	576	Word2Vec, LSTM layers: 2, Hidden units: 256, Embedding size: 300, Dropout: 0.2, Learning rate: 0.0001, Batch size: 64, Epochs: 20
BiLSTM		LSTM layers: [1,2]; Hidden units: [64,128,256]; Embedding size: [200,300]; Dropout: [0.1,0.2,0.3]; Learning rate: [0.0001,0.001]; Batch size: [32,64]; Epochs: [10,20]	576	FastText, BiLSTM layers: 2, Hidden units: 256, Embedding size: 300, Dropout: 0.2, Learning rate: 0.0001, Batch size: 64, Epochs: 20
BERT		Hidden layers: [1,2]; Hidden units: [128,256,512]; Dropout: [0.1,0.2,0.3]; Learning rate: [0.00001,0.0001]; Batch size: [16,32]; Epochs: [3,4]	144	Hidden layers: 2, Hidden units: 256, Dropout: 0.2, Learning rate: 0.00001, Batch size: 32, Epochs: 4

#### Comparison results

Tables 10 and 11 illustrate the comparative performance of the BERT-GCN-based method against various machine learning and deep learning models using the test dataset. According to Table 10, the BERT-GCN method achieved superior overall classification results, attaining the highest average F1 score (85.46%) and accuracy (85.23%) across all eight defect categories. Notably, in the more challenging categories, such as Class 3 and Class 8, BERT-GCN significantly outperformed other methods (e.g., Class 3: BERT-GCN F1 score = 70.75%, ANN = 67.67%, SVM = 62.62%; Class 8: BERT-GCN F1 score = 69.99%, ANN = 63.41%, SVM = 67.66%), demonstrating superior robustness.

Table 11 compares the inference speeds of the evaluated models. The BERT-GCN method achieves a fast inference speed (0.027 s/instance), slightly slower than BERT (0.021 s/instance) and similar to other deep learning models (e.g., BiLSTM and LSTM, both at 0.022 s). Despite combining transformer and graph convolutional layers, BERT-GCN remains computationally efficient due to effective GPU acceleration and optimized embeddings, clearly outperforming traditional machine learning methods (e.g., RF: 0.025 s, SVM: 0.024 s).

**Table 10:** Comparison results of best model on test set.

Category	Class1	Class2	Class3	Class4	Class5	Class6	Class7	Class8	Average
Proposed method									
F1 score	91.28	90.52	70.75	90.21	90.34	90.29	90.29	69.99	85.46
Accuracy	91.32	90.31	73.07	90.27	90.11	90.05	89.78	66.96	85.23
ANN									
F1 score	86.94	82.51	67.67	77.7	76.97	81.46	82.38	63.41	77.38
Accuracy	86.34	83.53	61.05	76.92	75.24	79.7	81.85	66.41	76.38
SVM									
F1 score	87.26	84.43	62.62	79.28	77.92	80.53	82.73	67.66	77.80
Accuracy	86.81	83.19	61.04	78.97	76.32	80.45	84.44	66.47	77.21
DT									
F1 score	85.34	82.52	60.85	76.75	74.3	79.72	81.32	63.33	75.52
Accuracy	85.65	82.33	60.16	77.02	74.52	79.33	80.44	66.12	75.70
RF									
F1 score	89.17	85.96	62.24	79.9	77.44	81.13	83.31	64.16	77.91
Accuracy	87.65	85.84	62.3	79.82	77.57	81.13	82.14	66.72	77.90
LR									
F1 score	87.65	85.17	64.78	74.29	71.58	83.41	82.5	40.63	73.75
Accuracy	87.54	84.38	63.67	73.35	71.37	82.33	82.48	40.21	73.17
1D CNN									
F1 score	89.75	83.99	63.02	80.1	78.5	81.52	86.18	65.8	78.61
Accuracy	89.06	84.6	63.3	79.07	77.57	81.13	83.8	65.02	77.94
LSTM									
F1 score	89.28	84.87	63.52	80.2	78.8	82.42	84.56	66.02	78.71
Accuracy	86.97	85.61	63.47	78.88	77.96	82.31	73.49	66.51	76.90
BiLSTM									
F1 score	89.58	85.97	63.72	81.52	78.93	82.62	84.96	66.82	79.27
Accuracy	86.97	85.71	63.97	79.25	78.1	82.51	84.35	67.1	78.50
BERT									
F1 score	90.28	86.87	66.52	83.92	81.53	88.57	87.56	68.02	81.66
Accuracy	90.17	86.61	65.07	83.57	81.3	88.42	87.23	67.9	81.28

**Table 11:** Detection speed by each method.

Method	Detection speed (seconds per instance)						
	Mean	Std	Min	25%	50%	75%	Max
Proposed method	0.027	0.001	0.026	0.026	0.026	0.027	0.028
ANN	0.023	0.002	0.020	0.022	0.023	0.024	0.028
SVM	0.023	0.002	0.021	0.023	0.024	0.025	0.029
DT	0.024	0.002	0.021	0.023	0.024	0.025	0.030
RF	0.025	0.002	0.021	0.024	0.025	0.026	0.030
LR	0.023	0.002	0.020	0.022	0.023	0.024	0.029
1D CNN	0.021	0.002	0.018	0.020	0.021	0.022	0.026
LSTM	0.022	0.002	0.019	0.021	0.022	0.023	0.027
BiLSTM	0.022	0.002	0.019	0.021	0.022	0.023	0.027
BERT	0.021	0.002	0.018	0.020	0.021	0.022	0.026

## Conclusions

This study evaluated four GNN-based text classifiers—TextGCN, TextING, TensorGCN, and BERT-GCN—on 4,212 fire-door defect reports from 8,786 housing units, training 1,008 model variants with shared and method-specific hyperparameters. Defects were grouped as Class1–Class8 (Frame gap, Door closer adjustment, Contamination, Dent, Scratch, Sealing components, Mechanical operation components, Others). On the test set, the selected BERT-GCN achieved an average F1 score of 85.46% and accuracy of 85.23%, with ≈ 90% F1 for Class1/2/6/7 and lower scores for Class3 (70.75%) and Class8 (69.99%); validation–test differences were within ± 1%, and per-instance latency was ~ 0.026–0.027 s. Against 2,430 baseline models (ANN, SVM, DT, RF, LR, 1D-CNN, LSTM, BiLSTM, and BERT), BERT-GCN delivered the highest average F1 score and accuracy, indicating the value of modeling document–term and term–term relations in short, technical defect narratives.

These findings suggest that relational cues around component terms and actions drive reliable detection in the egress-critical categories (Class1, Class2, Class6, Class7), whereas diffuse or catch-all wording limits performance for Class3 and Class8. For operational protection, high-confidence predictions in Class1/2/6/7 can trigger same-day work orders, medium-confidence cases can be queued for rapid human review, and Class4/5 (dent, scratch) can be batched for planned maintenance; the measured speed supports near-real-time triage on inspection devices. For entry-point protection oversight, class outputs can populate governance metrics—time-to-repair for Class1–Class2, recurrence by contractor or floor, and hotspot maps of systematic issues—while Class3 and Class8 remain subject to mandatory human confirmation and benefit from refined taxonomies and synonym handling.

Despite strong results, several limitations remain. The dataset comes from a single company and a narrow inspector cohort within a limited time window; description quality varies with inspector training, which can affect label consistency. Because the approach is data-driven, performance depends on the quality and representativeness of the corpus, so portability to new contexts will hinge on similarity to the training data. The corpus was also translated from Korean to English before modeling, which may introduce linguistic variation that was not explicitly quantified.

Future research should validate the method on multi-source datasets spanning different contractors, periods, and inspector backgrounds to improve generalizability; evaluate cross-lingual robustness by training and testing on the original Korean text and additional languages using multilingual encoders, translation-robust procedures such as back-translation consistency, and lightweight adapters; and investigate multimodal extensions that fuse defect text with available images or drawings—either via late fusion baselines or a multimodal GNN—while reporting both accuracy and latency to clarify deployment trade-offs.

## Data Availability

The datasets used and/or analysed during the current study are available from the corresponding author on reasonable request.
